# Fluoride Alteration of [^3^H]Glucose Uptake in Wistar Rat Brain and Peripheral Tissues

**DOI:** 10.1007/s12640-017-9709-x

**Published:** 2017-02-27

**Authors:** Anna Rogalska, Katarzyna Kuter, Aleksandra Żelazko, Anna Głogowska-Gruszka, Elżbieta Świętochowska, Przemysław Nowak

**Affiliations:** 1 Department of Toxicology and Health Protection, School of Public Health in Bytom, Medical University of Silesia, Piekarska 18, 41-902 Bytom, Poland; 2 Department of Neuro-Psychopharmacology, Institute of Pharmacology, Polish Academy of Sciences, Smetna 12, 31-343 Kraków, Poland; 3 Department of Communal Hygiene and Sanitary Supervision, School of Public Health in Bytom, Medical University of Silesia, Piekarska 18, 41-902 Bytom, Poland; 4 Department of Biochemistry, Clinical Biochemistry Division, Medical University of Silesia, Jordana 19, 41-808 Zabrze, Poland

**Keywords:** Fluoride, Exposure, Glucose transporter, Glucose uptake, Brain, Rats

## Abstract

The present study was designed to investigate the role of postnatal fluoride intake on [3H]glucose uptake and transport in rat brain and peripheral tissues. Sodium fluoride (NaF) in a concentration of 10 or 50 ppm was added to the drinking water of adult Wistar rats. The control group received distilled water. After 4 weeks, respective plasma fluoride levels were 0.0541 ± 0.0135 μg/ml (control), 0.0596 ± 0.0202 μg/ml (10 ppm), and 0.0823 ± 0.0199 μg/ml (50 ppm). Although plasma glucose levels were not altered in any group, the plasma insulin level in the fluoride (50 ppm) group was elevated (0.72 ± 0.13 μg/ml) versus the control group (0.48 ± 0.24 μg/ml) and fluoride (10 ppm) group. In rats receiving fluoride for 4 weeks at 10 ppm in drinking water, [3H]glucose uptake was unaltered in all tested parts of the brain. However, in rats receiving fluoride at 50 ppm, [3H]glucose uptake in cerebral cortex, hippocampus, and thalamus with hypothalamus was elevated, versus the saline group. Fluoride intake had a negligible effect on [3H]glucose uptake by peripheral tissues (liver, pancreas, stomach, small intestine, atrium, aorta, kidney, visceral tissue, lung, skin, oral mucosa, tongue, salivary gland, incisor, molars, and jawbone). In neither fluoride group was glucose transporter proteins 1 (GLUT 1) or 3 (GLUT 3) altered in frontal cortex and striatum versus control. On the assumption that increased glucose uptake (by neural tissue) reasonably reflects neuronal activity, it appears that fluoride damage to the brain results in a compensatory increase in glucose uptake and utilization without changes in GLUT 1 and GLUT 3 expression.

## Introduction

Fluorine, an abundant element in the earth crust, readily reacts with other elements to produce salts known as fluorides. Ground water becomes contaminated at fluoride levels ranging from 1.0 to more than 600 mg/l depending on the area of study (WHO 2006; Brindha and Elango 2011). For humans, drinking water is the largest contributor to the daily fluoride intake although it is also present in vegetables, fruits, black tea, and fish bones. Thus, the consumption of food and beverages made with fluoridated water is one source of natural intake of this element, but consumption of ordinary drinking water is the main source. Fluoride is also found in dusts, industrial wastes, and burning of coal. Additional routes of exposure are inadvertent ingestion of toothpaste (young children) and use of fluoridated dental products (i.e., mouth rinses, dental floss) (Jha et al. 2011 **).**

Fluoride in low amount (0.5–1.5 mg/l) has remarkable prophylactic effects in inhibiting dental caries while at higher doses it causes dental and skeletal fluorosis. However, harmful effects of excess fluoride intake are also observed in soft tissues like liver, kidney, and brain. From animal studies, we have learned that fluoride crosses the blood–brain barrier and accumulates in the brain. Neurotoxicity caused by chronic fluoride exposure in rats results in a marked increase in oxidative stress, lipid peroxidation, and a decrease in the activity of antioxidant enzymes in discrete brain regions (Flora et al. 2009; Ranpariya et al. 2011). Also, alterations in activity of the neurotransmitters and their metabolism (e.g., decrease in nicotinic acetylcholine receptors or norepinephrine (NE) and 5-hydroxytryptamine (5-HT) increase) is associated with memory impairment that outlasts short-term fluoride in laboratory animals (Pereira et al. 2011). Consequently, neurological symptoms, including reduced intelligence quotient in children, cognition and memory impairment with decreased learning ability have been reported in fluoride-polluted areas (Choi et al. 2012).

Another biochemical hallmark of fluoride exposure in mammals is impairment in glucose metabolism (Perumal et al. 2013) which is the obligate energetic fuel for the central nervous system (CNS) as well as the only substrate able to completely sustain neural activity. The brain glucose level represents a net balance of glucose uptake from circulation), glucose metabolism to lactate and CO_2_, and glucose transport back to the circulation (Sandoval et al. 2008). Nutrients are transported across cellular membranes by specific glucose transporters (GLUTs) and monocarboxylate transporters (MCTs). The transport of glucose into mammalian cells is mediated by the 13 different GLUT proteins, of which GLUT1 and GLUT3 are most abundantly expressed in the brain (Jurcovicova 2014). In recent years, many studies have indicated that abnormalities in glucose metabolism may affect cognitive functions, e.g., patients with Alzheimer’s disease display a specific pattern of low brain glucose metabolism relative to healthy controls, with additional reduced expression of GLUT and insulin receptors in the brain (Mooradian et al. 1997; Cunnane et al. 2010). These findings implicate impaired insulin signaling in the cognitive deficits in Alzheimer disease (Steen et al. 2005). Moreover, it was recently shown that MCT1 function in oligodendrocytes is essential to prevent neurodegeneration, indicating the potential involvement of nutrient transporters in the development and progression of other neurological disorders as well (e.g., amyotrophic lateral sclerosis) (Lee et al. 2012).

Data on fluoride exposure and glucose metabolism impairments in mammals are rather fragmentary: (1) chronic fluoride exposure results in hyperglycemia accompanying classical symptoms of fluorosis, with diabetogenic effect of fluoride deriving from inhibition of key enzymes in glycolysis and in the Krebs cycle (Grucka-Mamczar et al. 2007); (2) a recent study also indicates that fluoride exposure lowers insulin secretion, consequent to a reduction in insulin mRNA and its secretion from beta-cells). This may account for increased blood glucose levels in fluoride intoxicated animals (Garzia-Montalvo et al. 2009); and (3) developmental fluoride exposure causes neuronal degeneration, decreased brain glucose utilization, and decreased the protein expression of GLUT 1 with concomitant impairment in learning and memory through adulthood (Jiang et al. 2014).

The current study was conducted in order to assess the effects of fluoride exposure on the [^3^H]glucose uptake in the brain of adult Wistar rats. Because other tissues are also essential in the overall schema of fluoride effects on glucose metabolic abnormalities, we additionally assessed [^3^H]glucose uptake by peripheral tissues.

## Materials and Methods

### Animals and Treatment

Adult (age, 8 weeks) male and female Wistar rats weighing 200–250 g were obtained from the University Animal Department (Katowice, Poland) and were housed in a well-ventilated room, at 22 ± 2 °C under a 12-h light to:12-h dark cycle (lights on 7:00 a.m. to 7:00 p.m.), and with free access to food and water. Starting at 9 weeks sodium fluoride (NaF), 10 or 50 ppm, was added to drinking water for 4 weeks (exposure time). Control rats consumed tap water. On the last day of exposure, rats were transferred for further experimentation.

All procedures were approved by the Local Bioethical Committee for Animal Care, Medical University of Silesia (permission no 62/2011 issued on 2011.09.14) and are in accord with principles and guideline described in the NIH booklet “Care and Use of Laboratory Animals.”

### Analysis of Blood Fluoride Level

Fluoride concentration in urine was measured directly with an ion selective electrode (Orion 94–09, Orion Research, MS, USA) after the addition of TISAB II (Orion Research).

### Analysis of Blood Glucose Level

On the last day of exposure, rats of all groups (control, NaF 10 ppm and NaF 50 ppm) were sacrificed and trunk blood was taken for plasma glucose and insulin analysis. All samples were promptly centrifuged, and the sera were separated and stored at −20 °C until the time of analysis. Blood glucose levels were measured by the glucose oxidase-peroxidase method with automated clinical chemistry analyzer - Optium Omega (Abbott, Alameda, USA). Data were expressed in milligrams per milliliter (Kitamura et al. 2009).

### Analysis of Blood Insulin Level

Commercially available enzyme-linked immunosorbent assay kits (ELISA) were used (according to the manufacturer’s instructions) to determine insulin levels (Insulin LND, Germany). The absorbance measurements for all samples were performed using the Universal Microplate Spectrophotometer (μQUANT BIOTEK Instruments, Inc., Winooski, VT, USA) at 450 nm. Assay sensitivity for insulin was 15 μg/l; the intra- and interassay CVs were −4.6 and 6.5%, respectively (Rakatzi et al. 2004).

### 6-[^3^H]-D-Glucose Uptake

On the last day of exposure, rats were injected i.p. with 6-[^3^H]-D-glucose (Amersham Radiochemicals, Pittsburgh, PA, USA; 41 Ci/mmol; 0.5 μCi/g BW). After 15 min, rats were sacrificed and the frontal cortex, hippocampus, striatum, and thalamus with hypothalamus were dissected. The lungs, kidney, spleen, stomach muscle, jejunum, visceral adipose tissue, liver, femoral muscle, heart muscle, atrium, aorta, femoral bone, skin, oral mucosa, tongue, parotid gland, incisors, molar tooth, and mandible also were taken as samples for determination of radioactivity in a liquid scintillation counter. Each tissue sample was weighed and placed in a 20-ml scintillation vial containing 1 ml of Soluene-350 (Packard Inc., Downers Grove, Ill., USA). Each vial was then tightly sealed and incubated at 37 °C for 48 h, to solubilize tissue. Later, scintillation cocktail (10 ml, Dimilume-30, Packard Inc., Downers Grove, Ill., USA) was added, and the vials were briefly vortexed before being placed in a scintillation counter (Liquid Scintillation Counter, DSA 1409, Wallac, Finland). Results are expressed as disintegrations per minute (DPM) per 100 mg of wet tissue (mean ± SEM) for each group (Konecki et al. 2006).

### Immunohistochemistry and Densitometric Analysis of GLUT 1 and GLUT 3

Immunostaining for GLUT 1 and GLUT 3 protein expression was carried out according to Kuter et al. (2011). At 12 weeks of age (4 weeks NaF exposure) rats were decapitated, the brain was rapidly removed, postfixed in cold 4% paraformaldehyde for 7 days, and cryoprotected in 20% sucrose solution in phosphate-buffered saline (PBS). The brains were sectioned (30 μm frontal sections) on a freezing microtome (AP = 2.52 mm from bregma according to Paxinos and Watson (2007)). Free-floating sections were incubated for 48 h at 4 °C in primary antibodies (anti-CB1 receptor, 1:1000; Sigma, Germany), rinsed in PBS, then incubated for 30 min in secondary antibodies (anti rabbit biotinylated, 1:200, Vector Laboratories, UK) and processed using an ABC-peroxidase kit (Vector Laboratories, UK) and 3,3′-diaminobenzidine as a chromogen. Stained sections were mounted on slides, dried, dehydrated, cleared in xylene, and cover-slipped in a Permount medium (Fisher Scientific, USA).

Two sections stained immunohistochemically for GLUT 1 and GLUT 3 protein were analyzed densitometrically in the region of striatum and frontal cortex. All sections were scanned together, digitalized, adjusted for brightness, and regions of interest outlined using Multi Gauge programme (FUJIFILM). Optical density (OD) for each area was then determined. Background signal was subtracted from each section separately, from the region of corpus callosum. Results are presented as mean of each OD/area^2^ minus background.

## Data Analysis

Two-way analysis of variance (ANOVA) and the post-ANOVA test of Neuman-Keuls were used to test differences between groups. A *p* value of 0.05 or less was used to indicate a significant difference.

## Results

### Fluoride Intake and Fluoride Plasma Assay

In the present work, we observed that control rats consumed an average of 170.3 ml/kg/24 h tap water, whereas rats treated with NaF 10 or 50 ppm consumed an average of 200.6 ml/kg/24 h or 215.7 ml/24 h, respectively—leading to the intake of 2.11 and 11.43 mg/kg/24 h of fluoride, respectively. After multiplication by 28 days (exposure time), the cumulative amounts of fluoride consumption were 59.8 mg/kg (10 ppm) and 320.04 mg/kg (50 ppm).

**Fig. 1:**
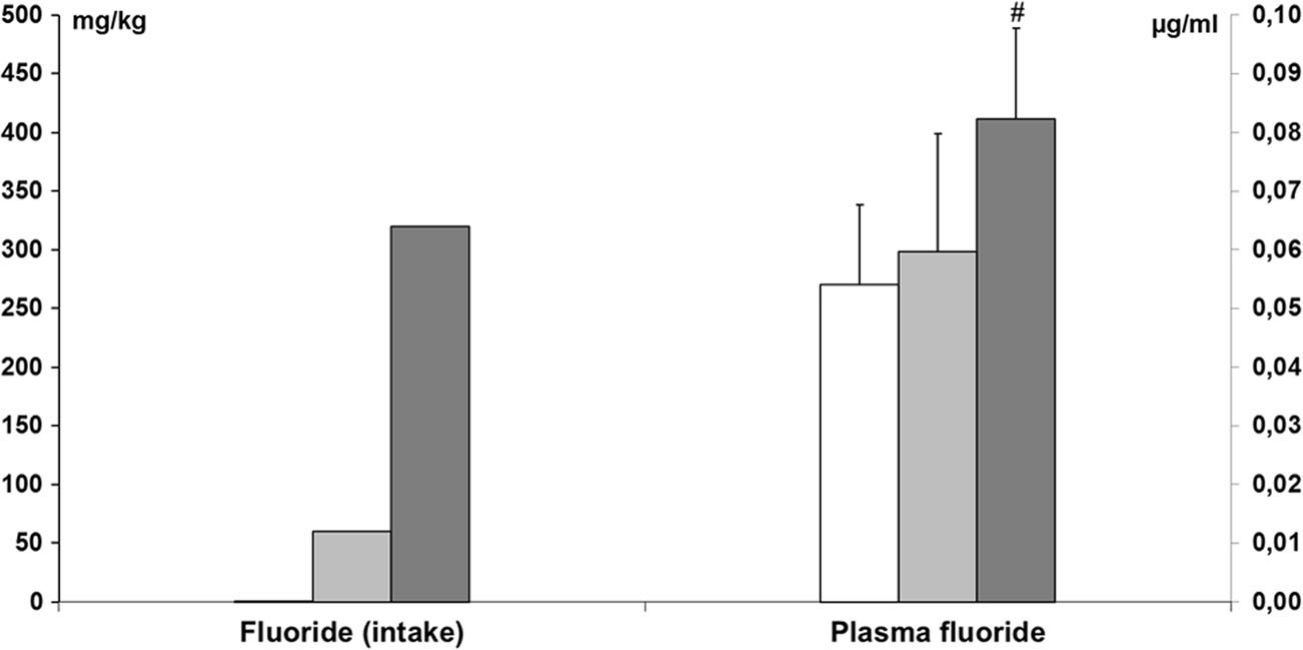
Fluoride intake and plasma fluoride concentration in adult rats exposed to NaF in drinking water in a concentration of 10 or 50 ppm (*n* = 8).  Control,  NaF 10 ppm,  NaF 50 ppm; # indicates *p* < 0.05

Plasma fluoride levels after 30 days of drinking fluoridated water were significantly (*p* < 0.05) higher in the group exposed to NaF 50 ppm (0.0823 ± 0.0199 μg/ml) in comparison to control (0.0541 ± 0.0135 μg/ml) and NaF 10 ppm (0.0596 ± 0.0202 μg/ml) (Fig. 1).

### Glucose and Insulin Blood Assay

**Fig. 2:**
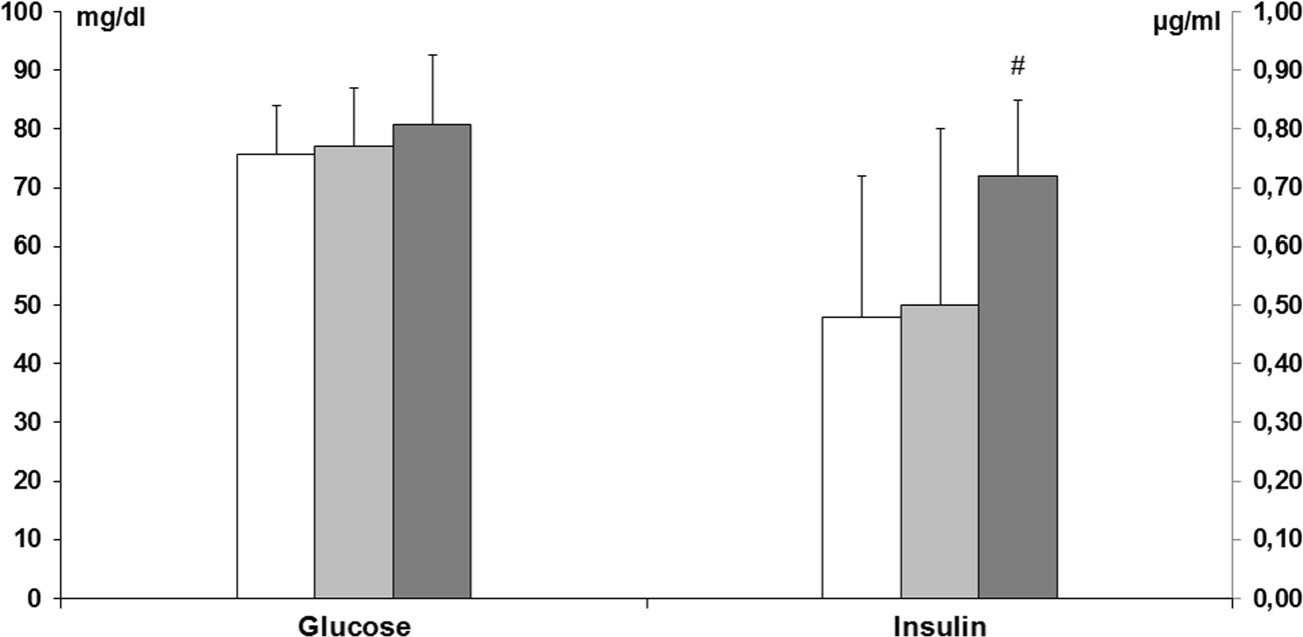
Glucose and insulin plasma concentrations in adult rats exposed to fluoride in drinking water in concentrations of 10 or 50 ppm (*n* = 8).  Control,  NaF 10 ppm,  NaF 50 ppm; # indicates *p* < 0.05

Plasma glucose concentration did not significantly differ among the experimental groups (ANOVA, *p* > 0.05), i.e., control—75.75 ± 8.17 mg/dl; fluoride (10 ppm)—77.13 ± 10.0; fluoride (50 ppm)—80.75 ± 11.88 mg/dl. However, plasma insulin levels were significantly higher as fluoride concentration increased in drinking water, attaining significance between control (0.48 ± 0.24 μg/ml) and fluoride (50 ppm) groups (0.72 ± 0.13 μg/ml) (ANOVA, Neuman-Keuls post-test, *p* < 0.05) (Fig. 2).

### [^3^H]Glucose Uptake in the Brain

**Fig. 3:**
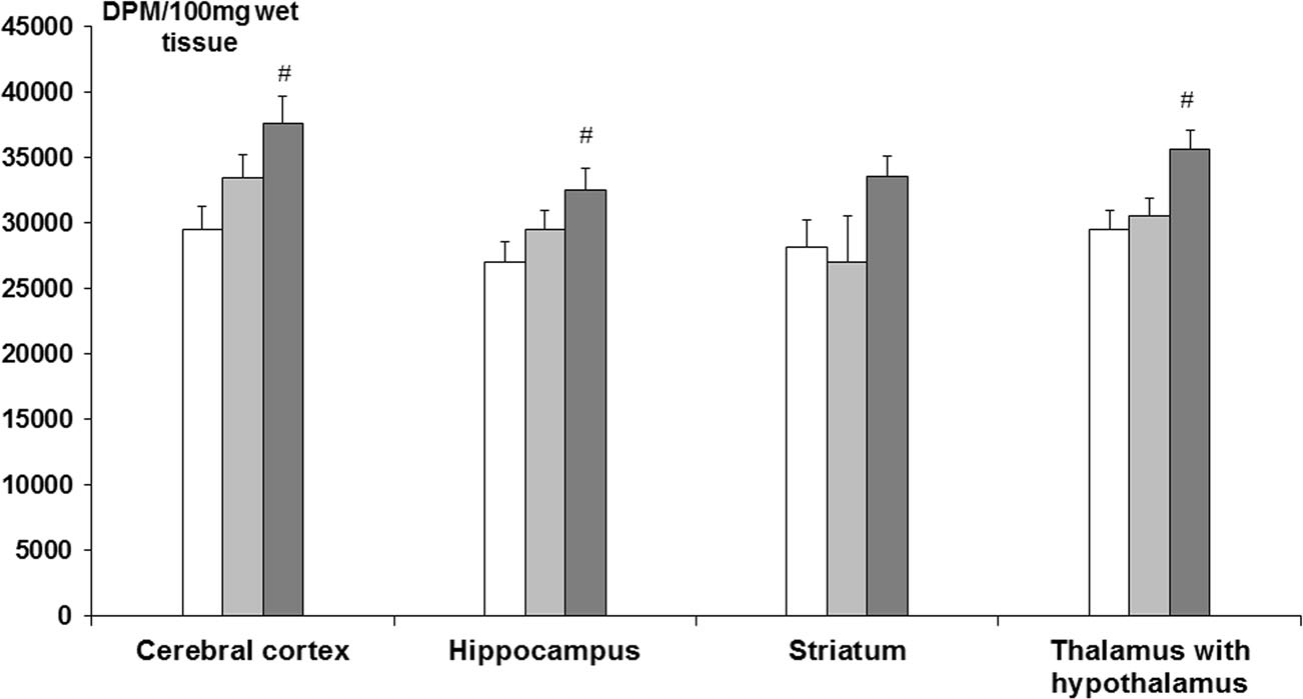
[^3^H]glucose uptake in the brain in adult rats exposed to NaF in drinking water, at a concentration of 10 or 50 ppm (*n* = 6).  Control,  NaF 10 ppm,  NaF 50 ppm; # indicates *p* < 0.05

In rats exposed to 10 ppm NaF, [^3^H]glucose uptake was unaltered in all tested parts of the brain. However, in rats exposed to 50 ppm NaF, [^3^H]glucose uptake in cerebral cortex, hippocampus, and thalamus with hypothalamus was elevated, versus the saline group (ANOVA, Neuman-Keuls post-test, *p* < 0.05) (Fig. 3).

### [^3^H]Glucose Uptake in Peripheral Tissues

NaF intake had negligible effects on [^3^H]glucose uptake by peripheral tissues (liver, pancreas, stomach, small intestine, atrium, aorta, kidney, visceral tissue, lung, skin, oral mucosa, tongue, salivary gland, incisor, molars, and jawbone). Only in rats exposed to NaF (10 ppm) there was a decrease in [^3^H]glucose uptake in thigh muscle and femoral bone (Table 1).

**Table 1:** [^3^H]glucose uptake in the in the peripheral tissues in adult rats exposed to NaF in drinking water, at a concentration of 10 or 50 ppm (*n* = 6)

Part of the body	Control DPM/100 mg of wet tissue	Postnatally exposed to NaF 10 ppm DPM/100 mg of wet tissue	Postnatally exposed to NaF 50 ppm DPM/100 mg of wet tissue
Liver	84,586.10 ± 7041.30	85,120.50 ± 7302.20	84,985.80 ± 2195.10
Pancreas	103,751.50 ± 19,052.90	110,152.00 ± 13,013.40	103,074.10 ± 6475.00
Stomach	45,109.90 ± 1279.40	47,682.80 ± 5522.30	47,153.50 ± 2787.40
Small intestine	60,244.10 ± 4025.50	56,374.40 ± 3603.50	65,553.00 ± 2200.50
Atrium	35,428.23 ± 1456.00	28,428.06 ± 2596.90	29,095.17 ± 2729.70
Heart muscle (ventricle)	35,575.80 ± 1321.00	37,726.30 ± 2051.70	36,792.90 ± 2071.20
Aorta	45,431.90 ± 4284.40	44,336.90 ± 3987.80	50,011.40 ± 2954.70
Kidney	89,556.50 ± 3259.50	91,945.90 ± 5389.70	98,784.80 ± 1809.00
Visceral fat	26,534.60 ± 5417.40	31,368.00 ± 4846.30	29,589.60 ± 5046.80
Lung	44,353.80 ± 2321.80	37,213.70 ± 4855.50	43,908.30 ± 2613.80
Muscle of thigh	28,771.30 ± 2351.40	20,402.20* ± 1721.60	26,897.70 ± 1353.00
Femur	23,858.20 ± 1483.30	17,299.00* ± 771.80	20,755.10 ± 1125.20
Skin	31,567.20 ± 764.90	30,097.40 ± 3451.60	35,249.80 ± 1072.70
Oral mucosa	41,683.40 ± 1354.40	37,149.20 ± 3539.40	37,397.30 ± 1524.20
Tongue	39,714.20 ± 1753.20	38,993.20 ± 2426.90	40,654.10 ± 1591.50
Salivary gland	29,322.20 ± 2467.20	29,490.80 ± 2047.40	31,732.80 ± 1556.60
Incisor	4056.20 ± 521.70	3462.70 ± 187.60	3714.20 ± 670.90
Molars	16,468.50 ± 1670.20	12,848.60 ± 1457.50	13,424.90 ± 908.80
Jawbone	15,937.20 ± 739.20	14,490.70 ± 853.00	16,008.60 ± 1174.00

*p < 0.05

### GLUT 1 and GLUT 3 in the Frontal Cortex and Striatum

**Fig. 4:**
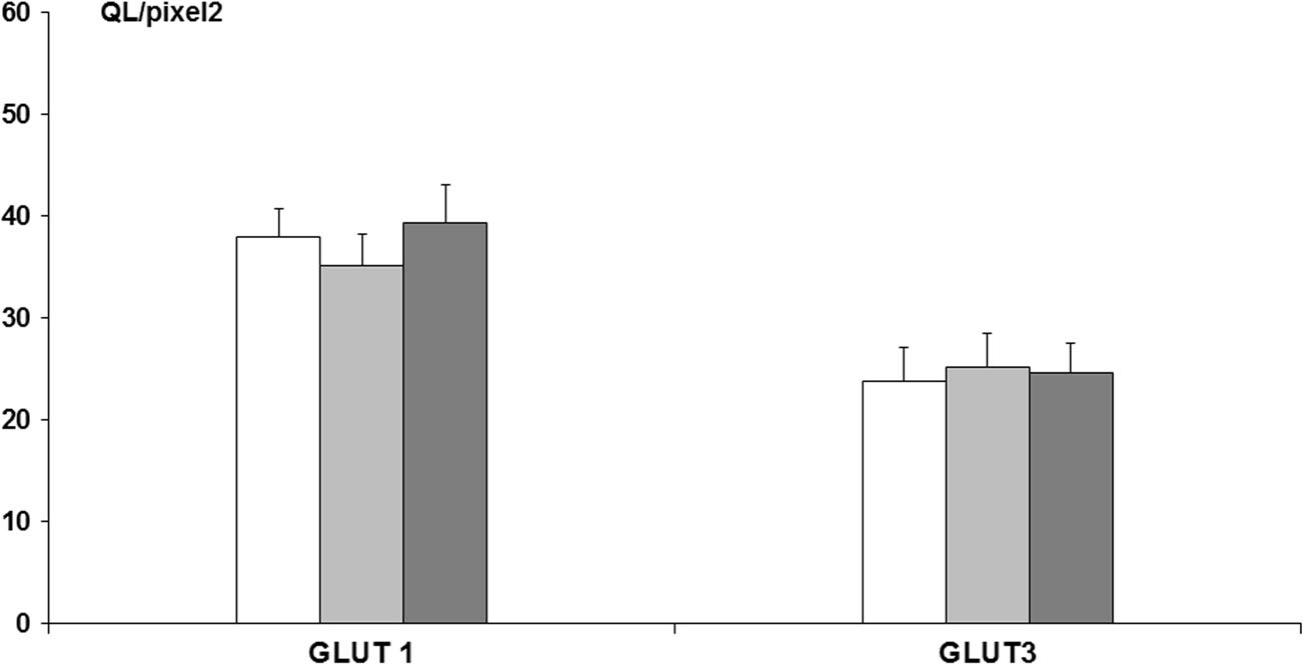
GLUT 1 and GLUT 3 protein immunoreactivity, assessed densitometrically in frontal cortex slices of adult rats exposed for 4 weeks to NaF in drinking water in a concentration of 10 or 50 ppm (*n* = 7).  Control,  NaF 10 ppm,  NaF 50 ppm; # indicates *p* < 0.05

Immunostaining for GLUT 1 and GLUT 3 protein expression was carried out in the frontal cortex, demonstrating that there were no significant changes among the experimental groups (ANOVA, *p* > 0.05), i.e., GLUT 1 (control—37.98 ± 2.71 QL/pixel^2^; fluoride (10 ppm)—35.13 ± 3.10 QL/pixel^2^; fluoride (50 ppm)—39.31 ± 3.08 QL/pixel^2^), GLUT 3 (control—23.80 ± 3.27 QL/pixel^2^; fluoride (10 ppm)—25.21 ± 3.25 QL/pixel^2^; fluoride (50 ppm)—24.60 ± 2.93 QL/pixel^2^) (Fig. 4).

**Fig. 5:**
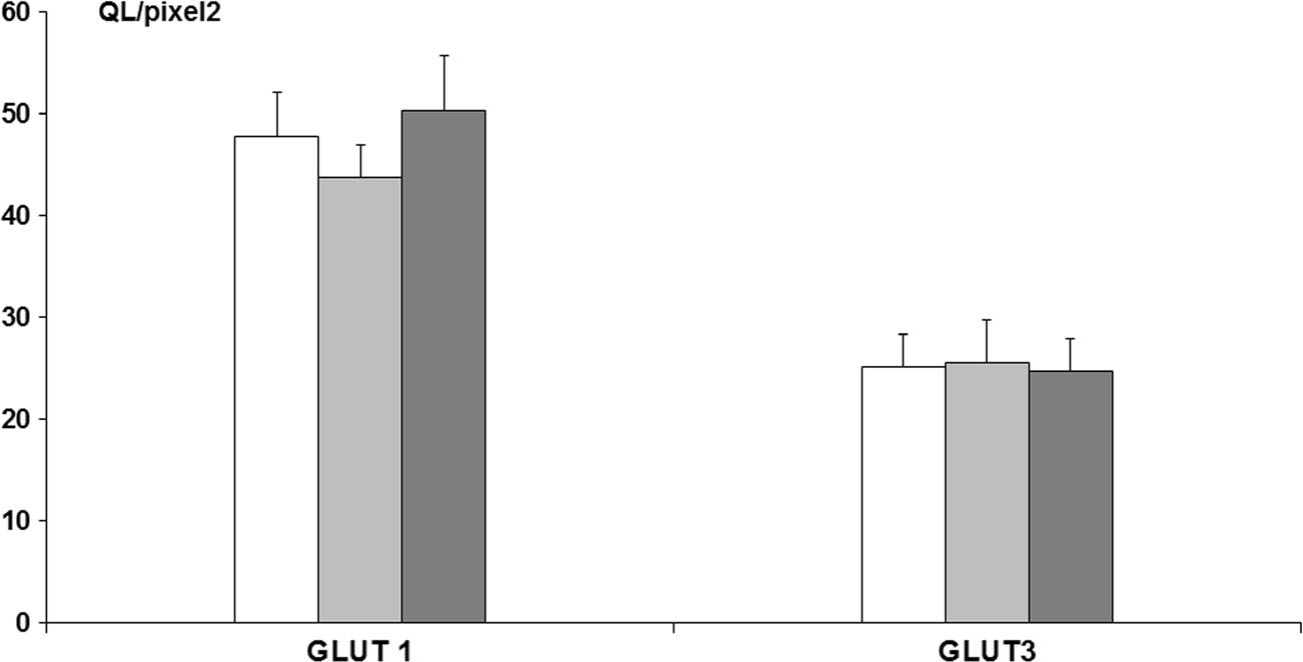
GLUT 1 and GLUT 3 protein immunoreactivity, assessed densitometrically in striatum slices of adult rats exposed for 4 weeks to NaF in drinking water in a concentration of 10 or 50 ppm (*n* = 7).  Control,  NaF 10 ppm,  NaF 50 ppm; # indicates *p* < 0.05

Immunostaining for GLUT 1 and GLUT 3 protein expression carried out in the striatum also showed no significant changes among the experimental groups (ANOVA, *p* > 0.05), i.e., GLUT 1 (control—47.80 ± 4.29 QL/pixel^2^; fluoride (10 ppm)—43.78 ± 3.21 QL/pixel^2^; fluoride (50 ppm)—50.21 ± 5.50 QL/pixel^2^), GLUT 3 (control—25.18 ± 3.22 QL/pixel^2^; fluoride (10 ppm)—25.57 ± 4.11 QL/pixel^2^; fluoride (50 ppm)—24.76 ± 3.10 QL/pixel^2^) (Fig. 5).

## Discussion

The main finding of the present study is that fluoride exposure, depending on the level of consumption, had qualitatively different effects on [^3^H]glucose uptake in the brain of rats. A low NaF concentration (10 ppm) failed to alter [^3^H]glucose uptake in all examined brain regions while a higher NaF concentration (50 ppm) increased [^3^H]glucose uptake, in the absence of change in GLUT 1 and GLUT 3 protein density in the brain. Alterations in [^3^H]glucose uptake by peripheral tissues were negligible in rats exposed to 10 or 50 ppm NaF in drinking water.

In contrast to peripheral effects of fluoride exposure, data on fluoride neurotoxicity in mammals are rather fragmentary and focused mainly on oxygen metabolism, the production of O_2_^−^ free radicals and activities of some antioxidant enzymes like superoxide dismutase (SOD), catalase and glutathione peroxidase (GPX) (Inkielewicz and Krechniak 2004; Shanthakumari et al. 2004). Notably, fluoride crosses the blood–brain barrier, to accumulate in hippocampal neuronal cell bodies and initiate a cascade of reactions which increases oxidative stress—the net effect being an elevation in malondialdehyde (the end product of lipid peroxidation) and an increase in damage to hippocampal neurons (Sharma et al. 2014). Bhatnagar et al. (2002) similarly demonstrated degenerated nerve cell bodies in the CA3, CA4, and dentate gyrus of hippocampus following NaF exposure in adult female mice. Thus, fluoride exposure, similarly to other neurotoxins, can cause overt damage to neuronal tissue. However, in addition to the effect of NaF in shifting the balance between free radical generation and antioxidant defense system, other mechanisms should be also considered, e.g., impairments in brain glucose metabolism—as we observed in the present work. Others have reported alterations in glucose uptake and utilization in the brain after other insults (Mehlhorn et al. 1998). Previously, we showed that in rats pre- and postnatally intoxicated with manganese (Mn 10,000 ppm) and lesioned as neonates with 6-hydroxydopamine (6-OHDA; an animal model of Parkinson disease) remarkable changes in [^3^H]glucose uptake in the brain were observed. More specifically, low dose 6-OHDA (2 × 15 μg icv) increased [^3^H]glucose uptake in brain. In rats lesioned with a moderate dose of 6-OHDA (2 × 30 μg icv), [^3^H]glucose uptake was unaltered in control and Mn exposed rats, while high dose 6-OHDA (2 × 67 μg icv) reduced [^3^H]glucose uptake in the brain of Mn exposed rats versus control (Kwieciński and Nowak 2009). On the premise that increased glucose uptake (by neural tissue) reflects neuronal activity, the damage by low dose 6-OHDA (mild insult) is the result of a compensatory increase in glucose uptake and utilization, while high dose of 6-OHDA with concomitant Mn (severe damage) overwhelms the regenerative ability of neurons and/or glia. It is likely that the type of changes (increase or decrease) as well as the intensity of glucose uptake disturbance depends on the severity of brain damage.

Because of a scarcity of data concerning the molecular mechanism of fluoride on brain glucose uptake, transport, and metabolism, it is difficult to account for the obtained effects of NaF. As previously stated, glucose levels in brain are a net balance between uptake, metabolism to lactate and CO_2_, and transport back to the circulation. Glucose enters cells mainly via GLUT 1 and GLUT 3, and several factors are able to modify GLUT protein expression and functioning. Handa et al. (2000) found in rats injected with ethanol (3 g/kg) that there was a decrease in GLUT 1 and GLUT 3 number but not in GLUT’s affinity in the brain (cortex). They also showed that there was no reduction in brain glucose utilization. Summing up, that study signifies that one must distinguish between glucose transporter protein expression and its affinity. In our study, immunostaining for GLUT 1 and GLUT 3 protein expression in the cortex and striatum was unaltered among the experimental groups (control; NaF 10 and 50 ppm). We hypothesize that elevated brain glucose uptake in rats exposed to NaF was brought about by an increase in GLUT affinity. Jiang et al. (2014) found that GLUT 1 protein expression was significantly decreased in the cerebral cortex and the hippocampus of both male and female rats treated pre- and postnatally with 25–100 mg/l NaF. By the small-animal PET/CT scan, they also revealed that the GLUT 1 expression corresponded with a low rate of glucose utilization in animals treated with higher doses of NaF. That is, NaF decreased the expression of GLUT 1, which then decreased brain glucose absorption and induced neuronal developmental toxicity. This report contrasts with our results but discrepancies may result from different doses of fluoride ingestion, a remarkably extended period of exposure (pre- and postnatal, approx. 12 weeks). In another ontogenetic study by Verma and Guna Sherlin (2002), there were reductions in serum levels of glucose in offspring of dams that were given NaF (40 mg/kg) orally from day 6 of gestation through the time of weaning at day 21. Conversely, Lombarte et al. (2013) demonstrated that in rats given NaF (15 mg/l) in drinking water there was a significant plasma increase in glucose level and insulin resistance. Others have shown an increase of serum insulin and a decrease of serum glucagon in rats treated with NaF (100 mg F/1 l) for 1 year (Hu et al. 2012). Summarizing, fluoride affects glucose and insulin levels in a dose and time dependent fashion—an increase or decrease can be observed. Thus, reported hyperglycemia may derive from an inhibitory effect of fluoride on the secretion of insulin, via altered intracellular signaling pathways related to insulin secretion (Menoyo et al. 2005). This effect was observed when plasma fluoride concentration was higher than 5 μM after a single dose of NaF in rats (7.6 mg F/kg body weight) and in humans (60 mg NaF) (Rigalli et al. 1990) and also in rats after administration of NaF (100 ppm) in drinking water for 30 days (Rigalli et al. 1992). The effect appears not to be related to insulin resistance (Chehoud et al. 2008), and the effect is absent when rats have high bone fluoride content, which is consistent with low plasma and tissue fluoride levels (Rigalli et al. 1992).

The brain is highly susceptible to oxidative stress because of high level of unsaturated fatty acids, high oxygen utilization, high iron content, and decreased activities of detoxifying enzymes (Bharath et al. 2002). Because glucose metabolism not only fulfills the energy requirement of the brain, but also provides ribose precursors for synthesis of nucleosides and NADPH which are necessary for synthesis of lipids and neurotransmitters as well as for the removal of free radicals (Nehlig 2004), we hypothesize the following “biological” sequence relating to fluoride exposure: initially, fluoride alters brain glucose metabolism which thus impairs energy balance and free radical clearance.

Despite the apparent direct adverse impact of fluoride on neuronal viability in the brain, a fluoride compensatory increase in glucose uptake and utilization may partially offset the neurodegenerative effects by enhancing the neuroprotective effects and/or regenerative ability of neurons and/or glia.
